# Challenges for Microelectronics in Non-Invasive Medical Diagnostics

**DOI:** 10.3390/s20133636

**Published:** 2020-06-29

**Authors:** Marco Carminati, Carlo Fiorini

**Affiliations:** 1 Politecnico di Milano, Dipartimento di Elettronica Informazione e Bioingegneria, 20133 Milano, Italy; carlo.fiorini@polimi.it; 2 Istituto Nazionale di Fisica Nucleare, Sezione di Milano, 20133 Milano, Italy

**Keywords:** ASICs, CMOS biosensors, lab-on-chip, multi-modal medical imaging, nuclear electronics, radiation detector, gamma cameras, SiPM

## Abstract

Microelectronics is emerging, sometimes with changing fortunes, as a key enabling technology in diagnostics. This paper reviews some recent results and technical challenges which still need to be addressed in terms of the design of CMOS analog application specific integrated circuits (ASICs) and their integration in the surrounding systems, in order to consolidate this technological paradigm. Open issues are discussed from two, apparently distant but complementary, points of view: micro-analytical devices, combining microfluidics with affinity bio-sensing, and gamma cameras for simultaneous multi-modal imaging, namely scintigraphy and magnetic resonance imaging (MRI). The role of integrated circuits is central in both application domains. In portable analytical platforms, ASICs offer miniaturization and tackle the noise/power dissipation trade-off. The integration of CMOS chips with microfluidics poses multiple open technological issues. In multi-modal imaging, now that the compatibility of the acquisition chains (thousands of Silicon Photo-Multipliers channels) of gamma detectors with Tesla-level magnetic fields has been demonstrated, other development directions, enabled by microelectronics, can be envisioned in particular for single-photon emission tomography (SPECT): a faster and simplified operation, for instance, to allow transportable applications (bed-side) and hardware pre-processing that reduces the number of output signals and the image reconstruction time.

## 1. Introduction

The impact of application specific integrated circuits (ASICs), in particular, of CMOS analog front-end interfaces for solid-state sensors and detectors, differs significantly among various application areas. For example, the field of high-energy and particle physics has been heavily reliant on integrated circuits for the readout of the detectors and parallel processing of signals for decades [1]. Instead, different from experimental physics, whose advancements are often constrained by the performance of electronics and instrumentation, in the field of bio-medicine, the role of microelectronics appears less consolidated nor univocal. In fact, despite a large penetration of micro-fabrication techniques in this field, and a considerable volume of publications, ASICs are rarely pivotal in the success of micro-analytical devices, especially from a commercial point of view.

**Figure 1:**
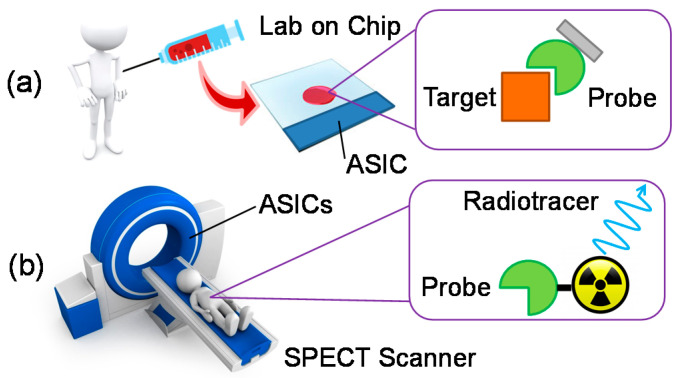
Complementary scenarios of the application of application specific integrated circuits (ASICs) in non-invasive medical diagnostics based on molecular specificity: (**a**) point-of-care analysis on a microfluidic chip and (**b**) single-photon emission tomography (SPECT) nuclear imaging in a clinical scanner.

In this brief tutorial review, we focus on non-invasive medical diagnostics and discuss some challenges for the future development of integrated electronics, with reference to diagnostics in particular. Non-invasive medical diagnostics comprise two complementary approaches: (i) imaging internal organs by means of energy penetrating through the skin and tissues, and (ii) taking out of the body a liquid sample for bio-chemical analysis (Figure 1). Non-invasive imaging can be performed by leveraging different mechanisms (providing different penetration depth, sensitivity, specificity, contrast, temporal and spatial resolution) such as: acoustic waves (ultrasonography), optical tomography, thermography, impedance tomography, X-ray photons (as in computed tomography (CT)), gamma-ray photons (as in positron emission tomography (PET) and single-photon emission tomography (SPECT)) and nuclear magnetic resonance imaging (MRI). Instead, the analysis of samples typically relies on the presence of specific markers, in a detectable concentration, within physiological fluids such as blood, urine, saliva, as well as sweat and tears. In particular, when looking for tumor markers, the latter is often called a liquid biopsy [2]. The marker (typically a protein) can be selectively identified by means of a matching molecule (of natural or synthetic origin) showing a strong bio-chemical affinity with it (such as enzymes, antibodies, nucleic acids and molecularly imprinted polymers). The conformational change induced by the binding of the receptor with the analyte is then converted into an electrical signal by means of several techniques. The most common ones are optical, either based on markers (fluorescence) or label-free (leveraging, for instance, the evanescent field at the core/cladding interface of a photonic waveguide), and magnetic or electrochemical (detecting the alteration of Faradaic or non-Faradic metal/electrolyte interfaces). 

The first approach is mostly based on sophisticated and expensive scanners, which require special facilities, the handling of radioactive material and skilled personnel, thus, are almost exclusively located in hospitals. The second approach, instead, is nowadays mostly pursued within the lab-on-a-chip (LoC) paradigm, aiming at the realization of portable and automatic micro-analytical platforms.

## 2. Lab-on-Chip Diagnostics

The main advantage offered by the deployment of CMOS ASICs in micro-analytical devices is miniaturization. Massively parallel readout systems (with thousands of channels, such as 1024 [3] or 2048 [4]) can be squeezed into a chip footprint of a few millimeters inside. The miniaturization of electronics matches the concurrent miniaturization of fluidics (LoCs handle volumes of samples in the microliter range) and of micro-machined transducers and electrodes. The main challenges which still need to be addressed in this area can be grouped into two classes: (i) at the circuit level (ii) at the level of packaging and interfaces. 

### 2.1. Circuit Challenges: Noise

At the circuit level, one consolidating trend is the reduction in power dissipation, both for thermal issues, critical in high-density chips, as well as the increase in the battery lifetime in the case of portable (and implantable) applications. In order to reduce the power dissipation in analog front-ends, different approaches can be adopted. Amplifiers can be shared among different circuit blocks [5] or stacked to re-use the same bias current [6]. Individual AC-coupled transistors can be stacked [7] and self-biased [8]. Analog processing (signal shaping, feature extraction) can be tailored to the type of signal and communication channel [9] and it is generally preferred to FPGA-based digital elaboration [10]. In order to reduce the energy consumption in data transmission, analog compression [11] and pulse-coded communication can be adopted [12]. A low-power design is typically opposite to a low-noise design, for instance, in terms of the flicker noise of MOSFETs, but is clearly fundamental in pushing down the limit of detection (LoD) of target molecules.

**Figure 2:**
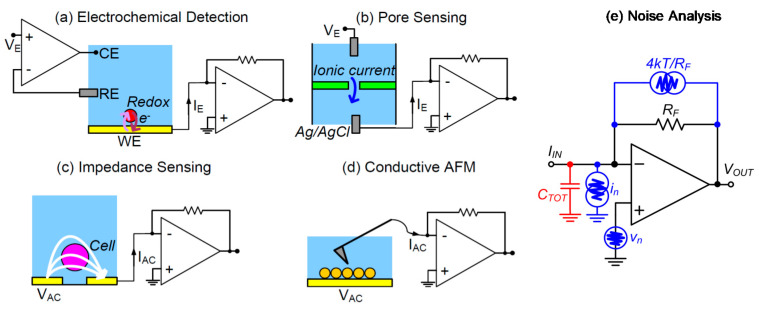
Examples of the application of a current front-end in bio-sensing for the: (**a**) redox detection of molecules, (**b**) current sensing molecule translocation through nanopores, (**c**) impedance detection, (**d**) nano-scale electrical probing, and (**e**) equivalent noise generators of the transimpedance amplifier (TIA).

In order to analyze the strategies and compromises in noise minimization, we consider the case of current amplifiers. Their application in bio-sensing is ubiquitous, spanning from electrochemical detection (where the charge is exchanged between the electrodes and redox molecules in the solution, Figure 2a) to optical detection (where the current is photo-generated in the photo-detector). As shown in Figure 2e, the input-referred current noise of a classical transimpedance amplifier (TIA) is given by the sum of three contributions: the thermal noise of the feedback resistor (*R_F_)*, the input-equivalent current noise of the amplifier (*i_n_*) and its voltage noise (*v_n_*). The latter becomes the dominant term in the total noise spectral density at high frequencies [13]. The voltage noise produces an input noise current proportional to the value of the total capacitance (*C_TOT_*) connected at the input. *C_TOT_* includes the feedback capacitance, the amplifier input capacitance, the sensor capacitance and the parasitic capacitance of the connection between the sensor/detector and the amplifier input. Evolutions of the basic TIA scheme, such as integrator–differentiator configurations, enable the combination of low-noise with extended detection bandwidth [13]. Additionally, for integrators (i.e., charge amplifiers) the minimization of the *C_TOT_* is crucial.

In order to minimize this noise contribution, three strategies can be adopted: (i) canceling the *C_TOT_* with an inductor, (ii) reducing the sensor capacitance, and (iii) reducing the parasitic capacitance. It has been shown that by placing an inductor parallel to the *C_TOT_* it is possible to improve the noise performance (of about one order of magnitude) thanks to the resonance [14]. This approach has two main limitations: (i) the enhancements are limited by the quality factor of the inductor, and (ii) the noise reduction takes place only in a narrow bandwidth around the resonance frequency (tens of MHz), thus being suitable only for impedance sensing at a fixed frequency.

A very relevant design guideline is the reduction in the capacitance associated with the sensor geometry, typically an electrode collecting the signal charge, whose area should be minimized. Of course, very often, the amount of collected charge is also proportional to the sensor areas, thus, in order to maximize the signal-to-noise ratio (SNR), noise should be minimized while preserving the signal amplitude. In the case of electrochemical sensors, despite a decrease in the working electrode area, the capture of molecules by means of this electrode should be simultaneously enhanced, with respect to passive diffusion, by means of active solutions, such as magnetic, electrophoretic, dielectrophoretic, thermal and fluid-dynamic ones. This approach proved to be very successful in the field of radiation detection, where the silicon drift detector (SDD) outperformed other solid-state detectors in terms of noise thanks to properly-shaped electric fields, which force the collection of the generated charge (across a wide depleted detection area) to drift towards a miniaturized anode [15]. Another drawback of shrinking the electrode area is the increase in the access impedance in the case of AC-coupled sensing, both in solid-state [16] and biological applications, such as impedance flow cytometry [17].

Another approach to reduce the sensor capacitance is the repartition of a large sensor area into N smaller ones, each one connected to an independent readout chain. In this way, the individual capacitance of each sensor is reduced by a factor of *N*. If the output signals of all *N* parallel chains are then summed, the signal will increase by a factor of *N* (i.e., it will recover a value equivalent to the case of a large area), while the noise (summed in power, since it is uncorrelated among chains) will only increase by √*N*, thus leading to an improvement of the SNR of √*N*. For example, this has been used in the readout of silicon photo-multiplers (SiPM) [18]. This interesting solution implies a growth in power and area occupation due to the multiplication of the readout chains. Furthermore, it is important to control the layout of connection between the sensor and the front-end: while the sensors capacitance decreases by a factor of *N*, the parasitic capacitance of the interconnection typically does not scale down and can become the dominant term.

**Figure 3:**
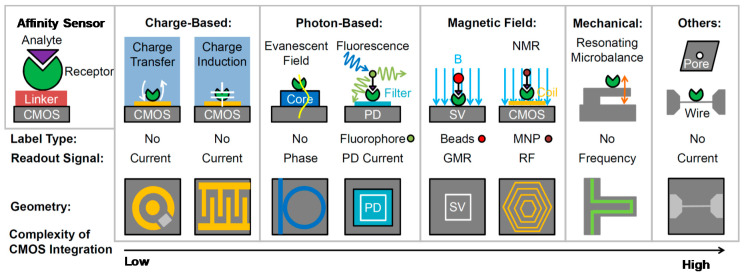
Overview of transducers (based on molecular affinity) on-chip for bio-sensing: electrical, optical, magnetic, mechanical and nano-scaled. The horizontal axis indicates the technological complexity of monolithic integration on CMOS substrates.

Finally, in order to fully profit from miniaturization and to preserve the detection performance by minimizing the length (and parasitics) of the interconnection, the transducers and the readout chip should be closely coupled and, when possible, monolithically integrated. From the noise point of view, the micro-sized sensor-on-chip solution is optimal [19]. Figure 3 shows a broad range of bio-sensing technologies that have been integrated on CMOS platforms: electrical and electrochemical, photonic, magnetic, micromechanical and nano-fabricated. Their position along the bottom horizontal axis qualitatively highlights the increasing technological complexity of integration on a CMOS substrate. RF electronics and on-chip coils even enable single-chip NMR and electron spin resonance detectors [20], targeting, in particular, point-of-care diagnostics [21]. Several efforts have been undertaken to improve the compatibility of bio-sensing with CMOS platforms: one example is the reduction in the temperature of fabrication of silicon nano wires (down to 200 °C), thus making them compatible with the standard CMOS process [22].

**Figure 4:**
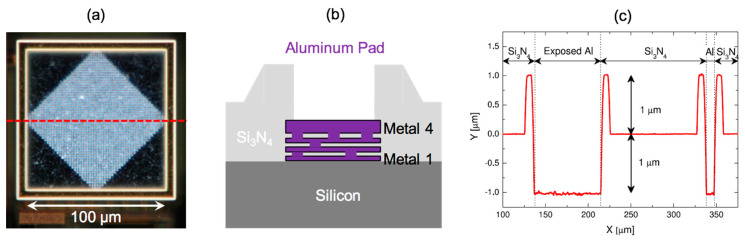
A dark-field micro-photograph of a standard Al bonding pad: (**a**) shows the cross-section, (**b**) shows the measured profile, and (**c**) highlights the shoulders of the passivation at the pad edges.

In the case of electrochemical sensing, the “electrode-on-chip” solution is apparently straightforward. The highest metal layer, commonly exposed to realize the bonding pads, can be used for patterning sensing electrodes. While this is simple in dry applications [23], it is more complicated in the case of bio-chemical sensing in liquid for two reasons: (i) materials and (ii) morphology. In fact, the metals commonly adopted to realize this metallization (aluminum and copper) are not suitable for electrochemical sensing, where noble metals (such as gold, silver and platinum) are preferred thanks to a stable (i.e., minimally reactive) behavior. Consequently, post-CMOS metallization is performed to electrochemically grow the proper metal on top of the working electrode. The morphology of the pad presents two additional issues: the roughness of the inner surface (even when avoiding the diamond of vias, visible in Figure 4a, typically realized to strengthen the stack of metal layer for wire bonding) is not negligible (~100 nm) and this inner area is surrounded by a 2 µm-tall shoulder due to the lifting of the top passivation layer (SiN) on the metal. Both issues can be solved, for instance, by evaporating a sensing area that extends on the side of the pad (on the flat nitride capping) as recently proposed [3]. 

To quantify the improvement in noise performance enabled by on-chip sensing, different figures of merit (FoM) can be adopted. For instance, in capacitive resolution, a *ΔC-FoM* can be defined at the input-referred noise spectral density in zF/√Hz, normalized on the amplitude of the forcing AC voltage signal. For dry sensing (detection of micro-particles in the air), the *ΔC-FoM* improved from 1100 zF·V/√Hz of a discrete-component implementation to 34 zF·V/√Hz of a monolithic integration [23]. Other figures of merit can include the power dissipation (tens to hundreds of mW for this application), area occupation, maximum operating frequency and detection bandwidth [19]. For measurements in liquid, we can consider the current resolution: in nano-pore current sensing, the current resolution (for a 100 kHz bandwidth) improves from 21.8 pA_RMS_ of the state-of-the-art discrete amplifier (with a miniaturized and cooled head-stage and integrator front-end with periodic reset) down to 12.9 pA_RMS_ with an integrated amplifier and on-chip electrode [24]. Taking as an example CMOS capacitive sensors, a steady improvement of the *ΔC-FoM* down to 1 zF·V/√Hz has been observed in the last decade [19,25], where the capacitance resolution has moved from the attoFarad to the zeptoFarad domains, achieved by miniaturization (parasitics reductions) and differential sensing [26] in the MHz range and leveraging resonance in the GHz range [27].

### 2.2. Packaging Challenges

**Figure 5:**
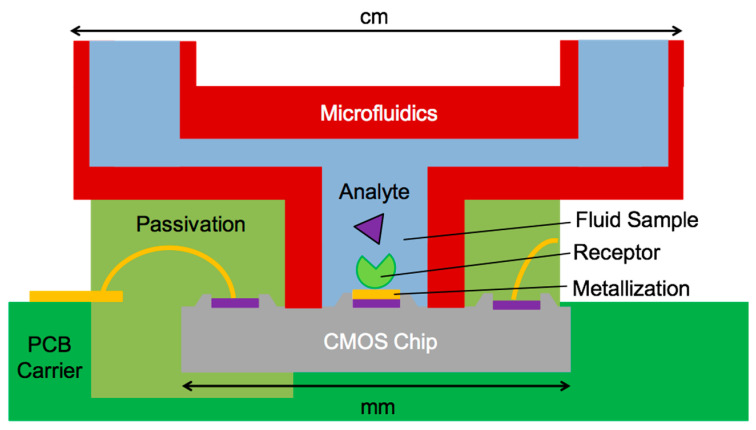
CMOS on-chip bio-sensing showing the critical size matching and post-fabrication steps: pad metallization, immobilization of receptor, coupling of mm-sized chips to cm-scale microfluidics and photo-patternable passivation to expose the sensing electrode.

Beyond the circuital and morphological challenges illustrated above, a pervasive diffusion of ASICs for biochemical detection is still hampered by additional technological challenges which include packaging, patternable passivation and the fluidic interface with the off-chip world. Different from all other application areas of microelectronics, here packaging has to protect the chip and its electrical interconnection (such as bonding wires) from environmental agents such as dust and humidity, while allowing the liquid sample to contact only specific areas of the chip with a spatial accuracy down to fractions of the pad size, i.e., to tens of micrometers (Figure 5). Packaging has to be reliable (to avoid leakage and aging during the device lifetime), bio-compatible (to avoid contamination of the biological sample) and cost-effective (since a very sophisticated packaging easily becomes the most expensive step of fabrication, as witnessed, for instance, in MEMS [28] and silicon photonics [29]). Coupling a CMOS ASIC to a microfluidic device is often complicated by the difference of materials (silicon vs. plastics, different metals) and disparity of sizes (mm-sized CMOS chips vs. cm-sized fluidics) [30]. A high-density placement of electrical and fluidic interconnect is typically achieved with a photo-patternable material deposited from the liquid phase, conformally covering bonding wires, which is cured and selectively removed to expose only the sensing electrode. The main challenge of this powerful approach lies in the preservation of the electrode surface cleanness (at the atomic level since the electrical double layer interfacial capacitance depends on the first ionic layer) after dry or wet etching of the passivation. A systematic review of the possible coupling strategies is reported here [31]. Sophisticated examples include both clean-room based post-CMOS approaches, achieving, for instance, planarization, gap filling of the CMOS chip embedded in a die carrier and coupled to taper joints [30], as well as lab-based solutions such as direct writing of a sacrificial channel [25].

In order to achieve cost-effectiveness, all these challenges should be addressed in a way compatible (in terms of production time and scalability) with large-volume industrial manufacturing. For this reason, polydimethylsiloxane (PDMS), which is the workhorse elastomer solution for laboratory-grade microfluidics, fabricated by means of replica molding and which offers excellent bio-compatibility, can hardly become a well-established industrial solution due to the cost and delicate steps in the fabrication process such as degassing and peel-off. Other polymers offering similar rapid prototyping versatility but a better resistance to chemicals and operation at high pressures have been identified [32]. Additive manufacturing (3D printing of plastic filaments or resins) [33], the micro-milling of rigid plastics (such as polycarbonate and PMMA) and laser ablation are all promising techniques for the fabrication of microchannels that, unfortunately, are all characterized by the sequential operation of a single machining head, not easily scalable to mass production. An emerging solution that can better suit the industrial manufacturability of biochips is represented by photo-patternable dry resists, such as SiNR [34]. Finally, the standardization of the design flow and of fluidic components and interconnects (similar to that of an electronic design) is still a chimera in this field and should be pursued in order to achieve market success in genomics and real-time, as well as point-of-care diagnostics, with the same effort that was devoted to the integration of the sample preparation on-chip.

## 3. Multi-Modal Medical Imaging

**Figure 6:**
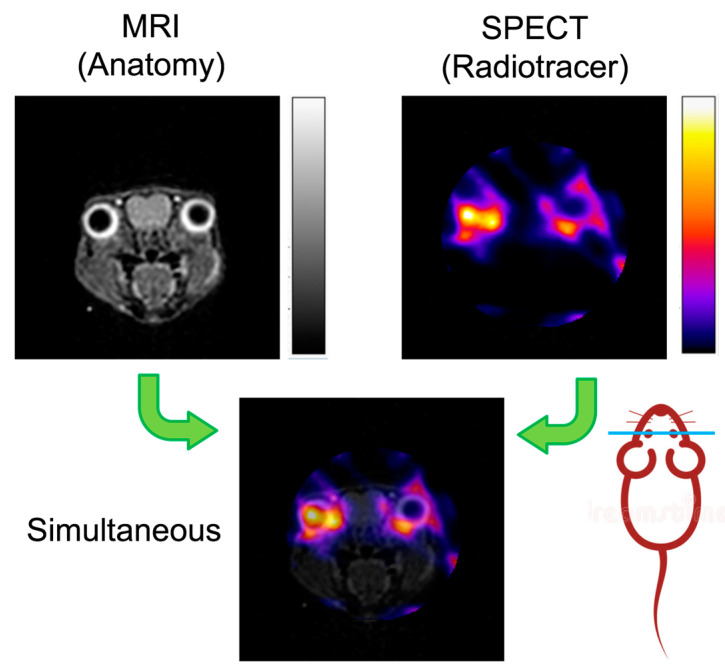
An example (15 mm-diameter axial slice of a mouse head) of multimodal imaging combining simultaneous magnetic resonance imaging (MRI (anatomical details)) with SPECT.

When the target molecule in the patient is not accessible from a fluid sample, nuclear imaging techniques with molecular selectivity are needed. The combination of different imaging techniques, offering complementary information, is becoming a consolidated trend in radiography. Typically, molecule-specific techniques (PET and SPECT) are combined with those providing anatomic information (such as CT and MRI) in order to register (i.e., align/overlay) both images (simultaneously acquired) and correctly locate the pathology in the body, as illustrated in Figure 6.

The main challenge posed by simultaneous imaging is clearly the mutual compatibility between the two scanning systems. Secondary challenges are the complexity and cost, to be compared with the clinical advantages (i.e., benefits for patients and clinicians, especially in terms of improved diagnostics and insight). Today, the adoption of integrated circuits is limited to the front-end of the acquisition chain in the gamma cameras pixels. We foresee that future breakthroughs in this field can be enabled by augmenting the range of functions implemented in silicon.

### 3.1. PET

PET is a nuclear imaging technique that relies on the emission of a pair of counter-propagating gamma photons (at an energy of 511 keV), generated by the annihilation of a positron with an electron [35]. The positron is produced by the decay of a radiotracer and this recombination takes places in the body within a short distance (~1 mm) from the radioligand. The major trade-off governing the performance of PET is between measurement time, sensitivity and injected activity [36]. There are two main development avenues. One is the extension of the axial field of view in order to improve the solid angle capturing gamma photons. Recently, the first results of a total-body PET have been reported [37]. The EXPLORER systems feature an axial field of view of 194 cm, extending along the whole body, and it is fully covered by 53,760 detection channels producing ~1 TB of data per scan. Thanks to a 40-fold increase in the signal with respect to traditional clinical scanners (capturing only a few percent of the emitted photons), the same SNR can be achieved by reducing the activity injected into the patient or by reducing the measurement time down to seconds. The latter result would lead to an unprecedented capability of tracking fast dynamics. Clearly, ASICs are pivotal in handling tens of thousands of channels and reducing the burden of data processing by implementing data pre-processing on-chip.

The second development direction is the use of the time-of-flight (ToF) information to improve the spatial resolution, sustained by the push to improve the timing resolution of photodetectors and readout electronics. A “10ps Challenge” has been launched [38] in order to stimulate step-changes in scintillator materials and detection approaches (looking, for instance, to prompt emissions), as well as in electronics [39]. Both perspectives clearly impact on the design of ASICs for PET: the first in terms of the number of channels and scalability and the second in terms of the intrinsic timing performance (currently in the ~100 ps range). If we take the coincidence time resolution (CTR_FWHM_) of a full detection system as a figure of merit to compare ASICs in terms of the timing performance (to be compared with similar scintillator and photodetector conditions), we can observe a continuous improvement trend. Considering LYSO crystals and 3 × 3 mm^2^ pixels, the TRIROC ASIC (2016 [40]) achieved 423 ps, TOFPET (2016) 294 ps and STiC (2018 [41]) 214 ps. For smaller pixels (2 × 2 mm^2^) FlexToT v2 reached 123 ps and NINO (2017) 93 ps [42].

### 3.2. SPECT

SPECT differs from PET in that it does not rely on proton annihilation: the radiotracer decays, directly emitting gamma-rays. In this case, a collimator is needed to select the trajectories of the photons impinging of the gamma camera. Tomographic images are then reconstructed from the planar projections imaged by the gamma cameras surrounding the patient. Different radiotracers can be employed, even simultaneously to map different molecules, if the energy resolution of the detector allows the discrimination of the photopeaks. The most commonly adopted is a metastable isotope of technetium (^99m^Tc) which has a line at 140 keV. Photons at such an energy are not absorbed within the thin silicon wafers and, thus, direct detection cannot take place. Instead, an indirect detection approach is adopted.

**Figure 7:**
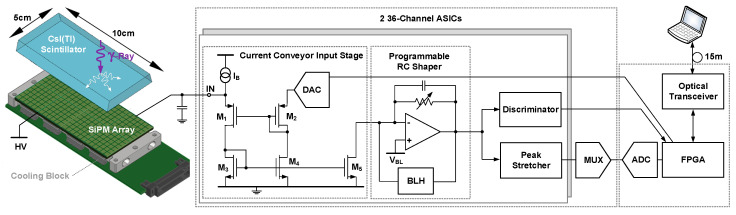
Architecture of the gamma camera of the INSERT SPECT system: the current signals of 72 silicon photo-multiplier (SiPM) pixels are read by two ASICs [43], featuring a low-impedance input stage, programmable shaper, peak stretcher and fast comparator to trigger the acquisition of events by the FPGA-based DAQ unit [44].

Figure 7 illustrates the typical architecture of a solid-state gamma camera. In particular, this scheme refers to the INSERT system which will be considered as a reference design. Each individual gamma photon is absorbed in the scintillator (here a CsI:Tl crystal of 8 mm thickness) producing a handful of visible photons, collected by the photodetectors placed at the bottom of the crystal. Given the limited number of photons, photodetectors with internal multiplication are commonly used. Previously, photo-multiplier tubes (PMT) were employed, but they are being systematically replaced by their solid-state equivalent: silicon photo-multiplers (SiPM). They offer a much higher compactness and, of utmost importance for MRI compatibility, much better compliance with magnetic fields. SiPMs are typically organized into tiles (here 6 × 6 pixels of 4 mm side in RGB-HD technology by Fondazione Bruno Kessler, Italy).

The current signal produced from a single SiPM can span from the µA to the mA range, with a typical duration in the tens of ns range. Given the large capacitance of the photodetector (~1 nF), differing from biosensors (typically in the pF range) and SDDs (in the sub-pF range), the additional parasitic capacitance of the interconnection (in the pF range) is not too critical, and the readout ASIC can be placed at a reasonable distance on the PCB. In order to grant a low input impedance, bandwidth and stability in these conditions, different topologies can be adopted. In the ANGUS ASIC [43], the weak positive feedback of a Themes current conveyor is successfully implemented. SiPMs are biased at ~35 V. The possibility to individually adjust the input potential with a DAC allows for the correction of gain mismatch across the channels (typically ~15%) and, more importantly, to selectively kill hot pixels affected by fault or large noise (dark counts). The tiles of SiPMs are typically cooled (here between 0 °C and −10 °C by refrigerant fluid) to reduce such a noise. The attenuated current pulses are then shaped by a programmable RC filter (~µs time constant), setting also the baseline. A fast discriminator triggers the acquisition of all channels and a peak stretcher allows the external 14-bit ADC to sequentially sample all outputs through a multiplexer. An FPGA acquires all the signals (at a rate of ~11 k frames per second) and transmits them to a laptop through an optical digital bus [44].

**Figure 8:**
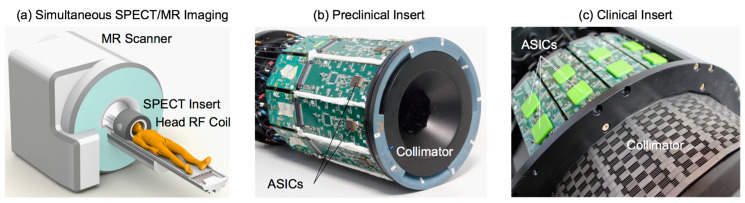
The INSERT family of MRI-compatible SPECT scanners featuring a static ring of gamma cameras: (**a**) shows the main concept of installation and components, (**b**) shows the preclinical system for small animals (10 modules), and (**c**) shows the clinical prototype for human brain studies (20 modules).

The operation of the INSERT system is shown in Figure 8a. The SPECT scanner is placed inside a standard MRI scanner, connected to a custom-designed and shielded transceiver coil placed inside the bore of the SPECT static ring, which contains gamma cameras and the collimator. Figure 8b shows the preclinical version of the scanner [45], while Figure 8c shows the clinical prototype [46], the only existing MRI-compatible SiPM-based SPECT insert for human brain imaging. The main features of the two instruments are summarized in Table 1.

## 4. SPECT Module Challenges and Outlook

### 4.1. Mutual Compatibility with MRI

Mutual compatibility between SPECT and MRI electronics entails several critical aspects. In order to reduce the distortion of the magnetic field due to the SPECT insert, electronic components (free of ferromagnetic metals such as nickel, present in connectors, plating pins and iron cores of inductors) and materials should be carefully selected. In particular, the introduction of metals in the MR bore should be minimized. For instance, collimators are fabricated by laminated layers or powder-epoxy mixtures to avoid solid regions and, thus, reduce the paths for Eddy currents. At an electronic level, it is crucial to reduce the irradiation of disturbances from the SPECT in the regions of the RF spectrum (~130 MHz). To minimize the impact of MRI on SPECT electronics, it is important to design special circuit geometries and layouts (avoiding loops and solid ground planes), add filtering on tracks that could pick-up RF interference and apply MRI-compatible shielding. The fundamental design guidelines and the results of compatibility tests are reported here [47].

### 4.2. Smaller, Faster Operation

Another direction of development at the system-level is to lighten and simplify the instrument and make it movable, in particular to make it a bed-side scanner. This would avoid the need to bring the patient to the room and might be relevant either during surgery (where intra-operative gamma probes are already used) or to promptly study sudden events such as epileptic seizures and strokes. Since the first movable MRI has been recently cleared by the FDA in the USA (model Lucy by Hyperfine, targeting stroke), a transportable SPECT/MRI system for the bed-side is foreseeable. The key to make a movable MRI system is in the use of low-field (and thus lighter) magnets [48]. The key to realizing a movable clinical SPECT is the reduction in the weight/size of the collimator, the detector refrigeration system (in the case of multiplication devices) and the electronics (again an opportunity for microelectronics to reduce the bulkiness and power dissipation in data processing). Additionally, the increase in speed by reducing the image-reconstruction time (currently in the hour time-frame, compared to acquisition times of about 15 min) is clearly related to new paradigms in the operation of SPECT, aiming to achieve quasi “real-time” imaging.

### 4.3. Integrated Processing

One of the major challenges hindering the scaling up of static preclinical SPECT systems to larger ones (i.e., with a larger field of view) is related to the growth in complexity in terms of the number of channels and signals travelling in the system (especially from a reliability point of view), and in terms of the processing time. One promising way to address both aspects is to shift the processing from the digital domain back to the analog domain, i.e., closer to the front-end. The reduction in the number of output channels can be achieved by using smart algorithms for machine learning (particularly for the estimation of the spatial coordinates of absorption of each gamma-ray photon) based on a partial readout of the crystal and by, for instance, principal component analysis and multiplexing strategies [49]. So far, they have been demonstrated in software post-processing and in discrete-hardware implementations (such as the decision-tree classification of gamma events embedded in a microcontroller [50]), but it is evident that an integrated implementation of such strategies in ASICs would offer significant benefits in terms of: (i) compactness, (ii) the robustness of signals and reliability, and (iii) processing time, as demonstrated by several emerging examples of hardware acceleration in image processing and machine learning based on neural networks on-chip [51,52,53].

### 4.4. Integration with Photonics

In order to increase the robustness of the signals through the long connection from the scanner to the base station placed in the control room, outside of the MRI room, and decouple them from electrical interferences, optical fibers are typically employed. In the case of the INSERT system, a 15 m digital optical daisy chain composed of two rings with 10 nodes each has been implemented. Since the operation of powerful digital processing platforms, such as FPGAs, inside the bore there can be severe issues of heat dissipation and mutual compatibility, it is possible to envision a tighter combination of readout ASICs with silicon-photonics transceivers, potentially on the same monolithic silicon platform as the robust analog transmission and direct modulation of thousands of optical signals injected in optical fibers, as recently proposed for high-energy physics [54].

## 5. Conclusions

We have summarized some recent results, development strategies and directions for integrated circuits and electronics for biomedical applications. Two apparently distant, but complementary, application areas in non-invasive diagnostics can both significantly profit from the advancements of CMOS technology. Targeted, yet cross-disciplinary, development of microelectronic designs and integration solutions can consolidate the pivotal role of integrated circuits in bio-sensing. Clearly, each application domain poses specific challenges and offers a very different performance of molecular detection: for example, the dynamic range of a micro-impedance sensing chip is 100–120 dB, while MRI can reach 150 dB at the expense of size, cost and measurement times that are several orders of magnitude larger. Some of the most relevant challenges have been discussed: packaging and the noise/power compromise in micro-analytical devices; scaling to a large number of channels; migration of processing from digital to analog, at least for the reconstruction of the position of the scintillation event; and transition towards transportable systems for medical imaging scanners. The impact of the fabrication cost significantly changes between the two application domains: when considering disposable diagnostic kits (with a production cost in the order of ~1$), the fabrication of silicon chips in CMOS foundries is economically sustainable only for mass production. Instead, when considering medical imaging scanners (with prices in the ~M$ range), small-volume and, thus more expensive, ASIC productions are still acceptable.

In conclusion, the current CoViD-19 pandemic has raised urgent concerns about diagnostic tools. The possible combination of CT scans of the chest with antibody-based assays for early, accessible and systematic diagnostics of the SARS-CoV-2 [55] is a vivid example of a possible effective combination of complementary diagnostic tools.

## Figures and Tables

**Table 1:** Summary of the INSERT performance.

Parameter	Preclinical [45]	Clinical [46]
Number of detection modules	10	20
Number of pixels	360	1440
Inner bore diameter [cm]	4	33
Outer insert diameter [cm]	20	50
Scintillator size [cm × cm × cm]	5 × 5 × 0.8	10 × 5 × 0.8
Energy resolution FWHM @140 keV	12%	16%
Extrinsic spatial resolution [mm_FWHM_]	0.9	10
Sensitivity [cps/MBq]	1105	365
Trans-axial Field of View [mm]	15.6	200
Collimator type	Multi-Pinhole	MM Slit-Slat
